# Higher blood pressure in adolescent boys after very preterm birth and fetal growth restriction

**DOI:** 10.1038/s41390-022-02367-3

**Published:** 2022-11-07

**Authors:** Jonas Liefke, Katarina Steding-Ehrenborg, Pia Sjöberg, Daniel Ryd, Eva Morsing, Håkan Arheden, David Ley, Erik Hedström

**Affiliations:** 1 Clinical Physiology, Department of Clinical Sciences Lund, Lund University, Skåne University Hospital, Lund, Sweden; 2 Paediatrics, Department of Clinical Sciences Lund, Lund University, Skåne University Hospital, Lund, Sweden; 3 Diagnostic Radiology, Department of Clinical Sciences Lund, Lund University, Skåne University Hospital, Lund, Sweden

## Abstract

**Background:**

Although preterm birth predisposes for cardiovascular disease, recent studies in children indicate normal blood pressure and arterial stiffness. This prospective cohort study therefore assessed blood pressure and arterial stiffness in adolescents born very preterm due to verified fetal growth restriction (FGR).

**Methods:**

Adolescents (14 (13–17) years; 52% girls) born very preterm with FGR (preterm FGR; *n* = 24) and two control groups born with appropriate birth weight (AGA), one in similar gestation (preterm AGA; *n* = 27) and one at term (term AGA; *n* = 28) were included. 24-hour ambulatory blood pressure and aortic pulse wave velocity (PWV) and distensibility by magnetic resonance imaging were acquired.

**Results:**

There were no group differences in prevalence of hypertension or in arterial stiffness (all *p* ≥ 0.1). In boys, diastolic and mean arterial blood pressures increased from term AGA to preterm AGA to preterm FGR with higher daytime and 24-hour mean arterial blood pressures in the preterm FGR as compared to the term AGA group. In girls, no group differences were observed (all *p* ≥ 0.1).

**Conclusions:**

Very preterm birth due to FGR is associated with higher, yet normal blood pressure in adolescent boys, suggesting an existing but limited impact of very preterm birth on cardiovascular risk in adolescence, enhanced by male sex and FGR.

**Impact:**

- Very preterm birth due to fetal growth restriction was associated with higher, yet normal blood pressure in adolescent boys.
- In adolescence, very preterm birth due to fetal growth restriction was not associated with increased thoracic aortic stiffness.
- In adolescence, very preterm birth in itself showed an existing but limited effect on blood pressure and thoracic aortic stiffness.
- Male sex and fetal growth restriction enhanced the effect of preterm birth on blood pressure in adolescence.
- Male sex and fetal growth restriction should be considered as additional risk factors to that of preterm birth in cardiovascular risk stratification.

## Background

Preterm birth and low birth weight both predispose for cardiovascular disease later in life.^1–3^ Biomarkers of cardiovascular disease, such as increased blood pressure and aortic stiffness, have been observed already in childhood and adolescence after preterm birth and low birth weight.^4,5^ However, improvements in perinatal management have decreased peri- and neonatal mortality and morbidity,^6–10^ and recent studies show normal blood pressure and arterial stiffness in young children born extremely preterm.^11–13^ These recent studies show no additive effect of birth weight deviation as a marker of fetal growth restriction (FGR) to that of preterm birth on blood pressure and arterial stiffness.^12,13^ Birth weight for gestational age is a crude marker of impaired fetal growth and includes constitutionally small but healthy infants. True FGR, on the other hand, can with high sensitivity be verified by abnormal fetal blood flow as measured by fetal Doppler velocimetry using structured clinical protocols.^14^ Further, whether one sex is more susceptible to the effect of preterm birth on blood pressure and arterial stiffness is not clear.^12,13,15^

We hypothesized that very preterm birth due to verified FGR is associated with elevated blood pressure and increased arterial stiffness in adolescence and that FGR exacerbates the effect of preterm birth. We secondly hypothesized that sex has a modifying effect on relationships between FGR, very preterm birth and cardiovascular outcomes. The current study therefore examined adolescents born very preterm due to FGR, verified with abnormal fetal blood flow velocimetry, using sensitive measures of blood pressure variations and aortic stiffness. 24-hour ambulatory blood pressure measurements (24-hour ABPM) is the preferred method for assessing blood pressure variations,^16^ and magnetic resonance imaging (MRI) is the reference standard to assess aortic stiffness using pulse wave velocity (PWV) and distensibility as surrogate markers.^17–19^

The specific aims were to assess the impact of very preterm birth and FGR on (1) 24-hour ABPM; (2) PWV and distensibility in the ascending and descending thoracic aorta using MRI; and (3) possible modifying effects by sex.

## Methods

### Study population and protocol

Examinations were performed at Skåne University Hospital, Lund, Sweden, between 2014 and 2019. The Regional Ethical Review board in Lund, Sweden, approved the study (Dnr 2013/244). All participants, and their guardians when appropriate, provided written informed consent before participation. Participants underwent 24-hour ABPM and MRI, and blood were sampled for measures of kidney function. Weight and height were measured in conjunction with 24-hour ABPM or MRI. Body surface area (BSA) was calculated using the Mosteller formula.^20^

**Fig. 1:**
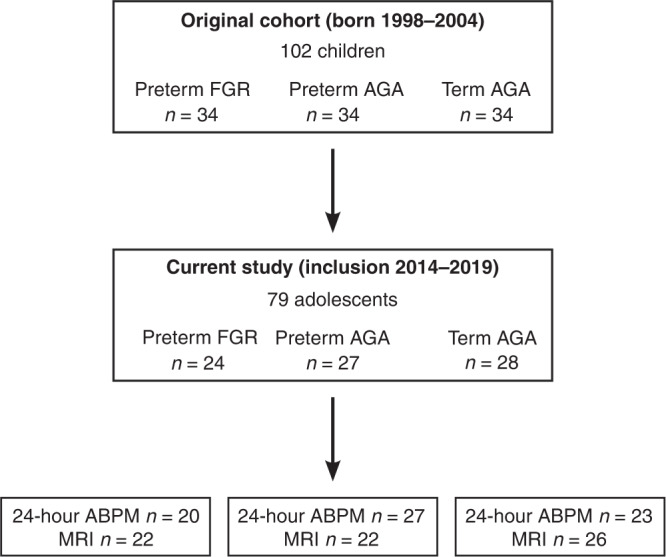
Inclusion flowchart. *Upper panel* shows initial inclusion of the original cohort. *Middle panel* shows the current follow-up in adolescence. *Lower panel* shows the number of individuals who underwent 24-hour ABPM and MRI. FGR fetal growth restriction, AGA appropriate for gestational age, MRI magnetic resonance imaging, 24-hour ABPM 24-hour ambulatory blood pressure measurements.

As part of a prospective cohort study, the current study included adolescents born very preterm with early onset FGR actively delivered due to fetal blood flow velocity abnormality between the years 1998 and 2004. Subjects had a birth weight <2 standard deviations,^21^ absent or reversed end-diastolic blood flow in the umbilical artery as determined by a standardized protocol using Doppler velocimetry, and were delivered with cesarean section between 24–29 gestational weeks (*preterm FGR*). Two control groups with birth weight appropriate for gestational age (AGA) born in the same time-period (1998–2004) were identified and a total of 102 participants were included prospectively and divided into three groups forming 34 matching triplets (Fig. 1). The first control group was a subset (*n* = 34) of all children (*n* = 371) who were born very preterm and who were admitted to the neonatal intensive care unit at Skåne University Hospital, Lund, Sweden. The causes of very preterm birth in this group included preterm premature rupture of the membranes, ablatio placentae, chorioamnionitis and being delivered as the healthy matched twin to a same sex individual in the preterm FGR group.^9^ This group was selected to match the preterm FGR group for sex, gestational age at delivery, and year of birth and thus consisted of those born very preterm without FGR (*preterm AGA*). Twins formed a matched pair if the following criteria were met; one twin was born with FGR, the other twin was born AGA, was of same sex, had normal blood flow in the umbilical artery, and twin-to-twin transfusion had been excluded.^9^ The second control group consisted of children born at term after normal pregnancy and were matched to both groups for sex and year of birth, forming the third triplet (*term AGA*). Detailed peri- and neonatal data and follow-up studies of cardiovascular, neuro-cognitive and pulmonary outcomes during childhood have been reported previously.^9,22–24^

All individuals from the original cohort were contacted via mail and by phone and asked to participate in this prospective cohort study in adolescence. Potential differences in the presence of peri- and neonatal confounders, e.g., maternal smoking, preeclampsia and bronchopulmonary dysplasia in those who participated versus those who opted out from follow-up were evaluated.

Preeclampsia was defined as diastolic blood pressure >90 mmHg on two or more consecutive occasions >4 h apart, arising after 20 weeks of gestation, and proteinuria >300 mg/L in two random clean-catch midstream urine specimens collected ≥4 h apart. Bronchopulmonary dysplasia was defined as need for supplemental oxygen (FiO_2_ >0.30) at an age corresponding to 36 gestational weeks.

### 24-hour ambulatory blood pressure measurement

24-hour ABPM was performed according to clinical routine. In short, systolic, diastolic, and mean arterial blood pressure were measured every 20 min over 24 hours, and activity and potential symptoms were reported. Daytime and nighttime periods were reviewed both separately and combined (SpaceLabs Medical ABP-monitor model 90207 or Ultralite TM 90217A, Issaquah).

Quality assessment and evaluation of the activity diary together with blood pressure readings was performed by a physician (PS) with 10 years of experience. Generally, >80% of successful readings was deemed sufficient for inclusion. Evaluation was in accordance with European Society of Cardiology (ESC) guidelines, using reference values for children and adolescents based on sex, age, and height.^16,25^ Mean systolic and diastolic blood pressures were graded as normal (<90th percentile), prehypertension (≥90th to <95th percentile) or hypertension (≥95th percentile) for daytime and nighttime separately. A nocturnal decrease in mean arterial blood pressure <10% was graded as pathological and in addition to reference values for children,^25^ systolic or diastolic blood pressures above adult reference values were graded as hypertension. Normal values for adults were defined for 24 hours as <130/80 mmHg, for daytime <135/85 mmHg, and for nighttime <120/70 mmHg.^16^

### Magnetic resonance imaging

Participants were imaged in supine position using a 1.5T MR scanner (Philips Achieva, Best, the Netherlands; or Magnetom Aera, Siemens Healthineers, Erlangen, Germany). Flow data were acquired in the ascending aorta and descending aorta at diaphragm level using a 2D phase-contrast gradient recalled echo sequence with retrospective ECG gating. Typical parameters were 35 timeframes per cardiac cycle, temporal resolution 23 ms, TR/TE = 9/6 ms, flip angle = 15°, 1.2 × 1.2 × 6 mm (Philips), and TR/TE = 10/3 ms, flip angle = 20°, 1.5 × 1.5 × 5 mm (Siemens). Velocity encoding was 150–250 cm/s to optimize for individual velocity resolution and to avoid aliasing. Respiratory-gated 3D angiography of the thoracic aorta was acquired using a T2-prepared balanced steady-state free precession sequence with isotropic resolution 0.88 mm (Philips) or 0.55 mm (Siemens).

Images were analyzed in Segment 3 (http://segment.heiberg.se, Medviso AB, Lund, Sweden).^26^ Two observers with 5 (JL) and 6 years (DR) of MRI experience performed analyses.

#### Vessel morphology

The ascending aorta and descending aorta at diaphragm level were delineated in magnitude images throughout the cardiac cycle using automatic segmentation with manual correction as needed, and guided by phase-contrast images where appropriate. Linear background phase correction was performed.^27^ Quantitative flow, flow curves, and cross-sectional area change throughout the cardiac cycle were analyzed.

#### Arterial stiffness

**Fig. 2:**
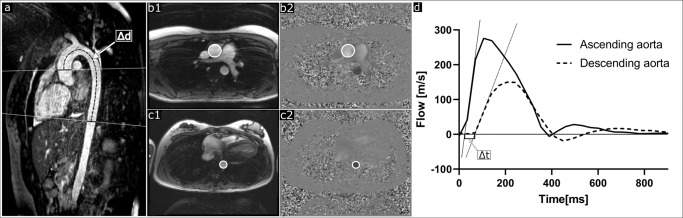
Measurement of pulse wave velocity in the thoracic aorta. **a** Shows non-contrast-enhanced 3D angiography of the thoracic aorta with flow measurement planes (solid lines) perpendicular to the ascending aorta and descending aorta at diaphragm level, and the aortic centerline distance (Δd; dashed line) between flow measurement planes. **b** Shows delineations of the ascending aorta in a magnitude image (b1) and phase-contrast image (b2). **c** Shows corresponding delineations of the descending aorta at diaphragm level (c1 and c2). **d** Shows flow curves for the ascending aorta (solid line) and descending aorta (dashed line) used to assess pulse wave velocity using the time-to-foot method. Pulse wave traveling time (Δt) was calculated as the time between upslope tangents intersecting the baseline. Pulse wave velocity was calculated by dividing the aortic centerline distance (Δd) with the time difference (Δt).

Aortic PWV was assessed as previously described,^28^ using the time-to-foot transit time method and blood pulse traveling distance by manual centerline 3D angiography between flow planes (Fig. 2), with flow-curve baseline correction.^19^

Thoracic aortic distensibility in the ascending aorta and descending aorta at diaphragm level was calculated as $\frac{{A_{max} \,-\, A_{min}}}{{A_{min} \,\cdot\, \Delta P}}$, where A_max_ and A_min_ are the maximum and minimum cross-sectional areas during the cardiac cycle, and $\Delta P$ equals the brachial blood pressure difference between systole and diastole.^29^ Oscillometric brachial blood pressure was acquired immediately after the respective flow acquisition.

### Cystatin C

Blood samples were collected in EDTA test tubes and directly centrifuged (Thermo Scientific Megafuge 8, Thermo Fisher Scientific, Waltham) at 1500 G for ten minutes, pipetted (Eppendorf Research plus 100–1000 μL, Hamburg, Germany) into cryotubes, and stored at −80 °C (Panasonic Ultra-low Temperature freezer, MDF-DU702VH-PE, Panasonic Healthcare Co., Ltd, Tokyo, Japan). Cystatin C was measured at Skåne University Hospital Clinical Chemistry laboratory using validated routine clinical assays. Estimated glomerular filtration rate (eGFR) was calculated using the Caucasian, Asian, Pediatric and Adult (CAPA) equation.^30^

### Statistical analyses

Statistical analyses were performed using SPSS 26.0 (IBM Corp, Armonk, New York) and GraphPad Prism 9 (GraphPad Software, La Jolla, California). Artwork was performed using the free and open-source vector graphics editor Inkscape (https://inkscape.org/). For 24-hour ABPM data, boys and girls were analyzed separately as per clinical routine whereas MRI data were analyzed both with both sexes combined and separately. Data are expressed as median (range). Kruskal-Wallis with Bonferroni’s multiple comparison test assessed group differences. The Jonckheere-Terpsta and Kendall’s Tau-b tests assessed trends between groups. Pearson’s chi-squared test or Fisher’s Exact Test assessed categorical variables. *P* values <0.05 were considered to show statistically significant differences.

## Results

### Study population

A total of 79 adolescents agreed to participate in the current study (14 (13–17) years, 52% girls). Figure 1 shows a flowchart of inclusion and Table 1 shows subject characteristics at birth and in adolescence as well as neonatal morbidity. In adolescence, when stratifying for sex, girls born preterm FGR were shorter than girls born term AGA (157 vs. 164 cm; *p* = 0.003). Of those who underwent pharmacological treatment for persistent ductus arteriosus (PDA), 2/10 individuals in the preterm FGR group and 3/9 individuals in the preterm AGA group also underwent surgical treatment for closure of the PDA while one individual in the preterm FGR group underwent surgical treatment alone (Table 1). Data from the follow-up study in childhood for the study population included in the current study show that there were no differences in parents’ educational level between the preterm FGR, preterm AGA or term AGA groups, with 22, 25 and 26 of the mothers (*p* = 0.99) and 23, 25 and 27 of the fathers (*p* = 0.80) having gone through upper secondary school education. The respective numbers for university education were 11, 13 and 13 for mothers (*p* = 0.99) and 7, 8 and 10 for fathers (*p* = 0.80). The number of families who were cohabited at time of follow-up in childhood (~7 years) were 18, 18 and 25 for the respective group (*p* = 0.10).^22^

**Table 1:** Subject characteristics and neonatal morbidity.

				*P* values			
				Between	Preterm FGR vs.	Preterm FGR vs.	Preterm AGA vs.
	Preterm FGR	Preterm AGA	Term AGA	Groups	Preterm AGA	Term AGA	Term AGA
Peri-and neonatal characteristics	*n* = *24*	*n* = *27*	*n* = *28*				
Gestational age at birth (days)	188 (172 to 204)	193 (171 to 208)	280 (269 to 285)	**0.0001**	>0.99	**0.0001**	**0.0001**
Gestational age at birth (weeks + days)	26 + 6 (24 + 4 to 29 + 1)	27 + 4 (24 + 3 to 29 + 5)	40 + 0 (38 + to –40 + 5)	**0.0001**	>0.99	**0.0001**	**0.0001**
Birth weight (g)	643 (395 to 976)	1100 (660 to 1790)	3485 (2850 to 4390)	**0.0001**	**0.003**	**0.0001**	**0.0001**
Birth weight deviation (%)	–34.6 (−62.7 to −22.5)	−3.6 (−23.3 to 14.3)	−1.6 (−16. to –28.8)	**0.0001**	**0.0001**	**0.0001**	>0.99
Maternal age (years)	33 (20–42)	31 (17–41)	31 (21–41)	0.2			
Preeclampsia (*n* (%))	8 (33%)	1 (4%)^#^	0	**<0.01**^**a**^			
Antenatal steroid treatment (*n* (%))	23 (96%)	26 (100%)^#^	0	0.5^a^			
Multiple birth (*n* (%))	5 (21%)	6 (22%)	0	>0.99^a^			
Cesarean section (*n* (%))	24 (100%)	15 (56%)	0	**<0.0001**^**a**^			
Smoking in pregnancy (*n* (%))	2 (8%)	6 (22%)	3 (11%)	0.3			
Ablatio placentae (*n* (%))	0	3 (11%)^##^	0	0.2^a^			
Neonatal morbidity							
Severe IVH grade III–IV (*n* (%))	2 (8%)	3 (11%)	0	>0.99^a^			
Any grades of IVH (*n* (%))	3 (13%)	7 (26%)	0	0.3^a^			
Bronchopulmonary dysplasia (*n* (%))	18 (75%)	7 (26%)	0	**0.001**^**a**^			
Severe retinopathy of prematurity (*n* (%))	2 (8%)	3 (11%)	0	>0.99^a^			
Necrotizing enterocolitis (*n* (%))	2 (8%)	0	0	0.2^a^			
Surgical treatment for PDA (*n* (%))	3 (13%)	3 (11%)	0	0.6^a^			
Pharmacological treatment for PDA (*n* (%))	10 (42%)	9 (33%)	0	0.5^a^			
Septicemia (*n* (%))	13 (54%)	6 (22%)	0	**0.02**^**a**^			
Asphyxia; Apgar score <7 at 5 min (*n* (%))	1 (4%)	6 (22%)	0	0.1^a^			
Periventricular leukomalacia (*n* (%))	1 (4%)	1 (4%)	0	>0.99^a^			
Inotropic support (*n* (%))	10 (42%)	8 (30%)	0	0.4^a^			
Surfactant treatment (*n* (%))	13 (54%)	15 (56%)	0	>0.99^a^			
Postnatal steroid treatment (*n* (%))	9 (38%)^##^	4 (15%)^#^	0	0.06^a^			
Ventilatory support at 36 GW (*n* (%))	4 (17%)	2 (7%)	0	0.4^a^			
Characteristics in adolescence							
Age (years)	15 (13–17)	15 (13–16)	15 (13–16)	0.9			
Weight (kg)	51 (30–90)	56 (37–75)	58 (37–89)	0.1			
Weight deviation (SD)	−1 (−4.6–2.9)	0.1 (−3–1.5)	0.4 (−2.3–2.6)	0.07			
Length (cm)	160 (150–180)^**1**^	167 (149–183)	167 (155–189)	**0.01**	**0.03**	**0.02**	>0.99
BMI (kg/m²)	20 (13–28)	21 (15–24)	21 (15–25)	0.9			
BSA (m²)	1.5 (1.1–2.1)	1.6 (1.2–2.0)	1.7 (1.3–2.2)	0.06			
Systolic blood pressure (mmHg)	105 (87–123)	107 (88–120)	102 (89–130)	0.37			
Diastolic blood pressure (mmHg)	53 (41–74)	54 (45–65)	50 (44–80)	0.08			

Data are presented as median (range). Neonatal data missing in one (^#^) and two (^##^) individuals.

*FGR* fetal growth restriction, *AGA* birth weight appropriate for gestational age, *IVH* intraventricular hemorrhage, *PDA* persistent ductus arteriosus, *GW* gestational week, *N/A* not applicable, *SD* standard deviation, *BMI* body mass index, *BSA* body surface area.

^1^Girls born preterm FGR were shorter than girls born term AGA (157 (150–165 cm) vs. (164 (157–176 cm); *p* = 0.003). Bold indicates differences between groups.

^a^Comparison performed between preterm FGR and preterm AGA.

Table 2 shows comparisons of perinatal characteristics and neonatal morbidity between those who participated in the current study and those included in the original cohort who opted out from follow-up in the current study.

**Table 2:** Comparison of perinatal characteristics and neonatal morbidity between study participants in the current cohort (opted in) and those who opted out from follow-up (opted out) from the original cohort.

	Preterm FGR			Preterm AGA			Term AGA		
	Opted in	Opted out	*P*	Opted in	Opted out	*P*	Opted in	Opted out	*P*
	*n* = *24*	*n* = *10*		*n* = *27*	*n* = *7*		*n* = *28*	*n* = *6*	
Perinatal characteristics									
Girls (%)	54	33	0.3	52	29	0.4	50	33	0.7
Gestational days (days)	188	191	0.6	193	180	0.1	280	279	0.7
Birth weight (g)	643	660	0.8	1100	875	**0.04**	3485	3580	0.3
Birth weight deviation (%)	−34.6	−43.4	0.3	−3.6	−11.4	0.1	−1.6	−1.2	0.7
Maternal age (years)	33	28	**0.01**	31	32	0.4	31	29	0.2
Preeclampsia (%)	33	0.3	1	4	0^#^	1	0	0	N/A
Antenatal steroid treatment (%)	96	90	0.5	100	86^#^	0.2	0	0	N/A
Multiple birth (%)	21	30	0.7	22	14^#^	1	0	0	N/A
Cesarean section (%)	100	100	1	56	86^#^	0.2	0	0	N/A
Primipara (%)	67	40	0.3	44	60^#^	0.7	25	33	1
Smoking during pregnancy (%)	8	30	0.1	22	0^#^	0.3	11	33	0.2
Ablatio placentae (%)	0	0	N/A	12	57^#^	**0.03**	0	0	N/A
Neonatal morbidity									
Severe IVH grade III–IV (%)	8	10	1	11	29	0.6	0	0	N/A
Bronchopulmonary dysplasia (%)	75	60	0.4	26	43^#^	0.6	0	0	N/A
Severe retinopathy of prematurity (%)	8	30	0.1	11	14^#^	1	0	0	N/A
Necrotizing enterocolitis (%)	8	20	0.6	0	0	N/A	0	0	N/A
Surgical or pharmacological treatment for PDA (%)	46	40	0.8	33	43^#^	0.6	0	0	N/A
Septicemia (%)	54	50	1	22	14^#^	0.4	0	0	N/A
Asphyxia; Apgar score <7 at 5 min (%)	4	20	0.2	22	14^#^	1	0	0	N/A
Periventricular leukomalacia (%)	4	0	1	4	14^#^	0.4	0	0	N/A
Inotropic support (%)	42	1	0.1	30	57^#^	0.2	0	0	N/A
Surfactant treatment (%)	54	60	1	56	86^#^	0.2	0	0	N/A
Postnatal steroid treatment (%)	41	38	1	15	57^#^	**0.04**	0	0	N/A
Ventilatory support at 36 GW (%)	17	40	0.2	7	0	1	0	0	N/A

Neonatal data missing in one (^#^) individual.

*FGR* fetal growth restriction, *AGA* birth weight appropriate for gestational age, *IVH* intraventricular hemorrhage, *PDA* Persistent ductus arteriosus, *N/A* not appropriate, *GW* gestational weeks. Bold indicates differences between groups.

### Blood pressure

**Fig. 3:**
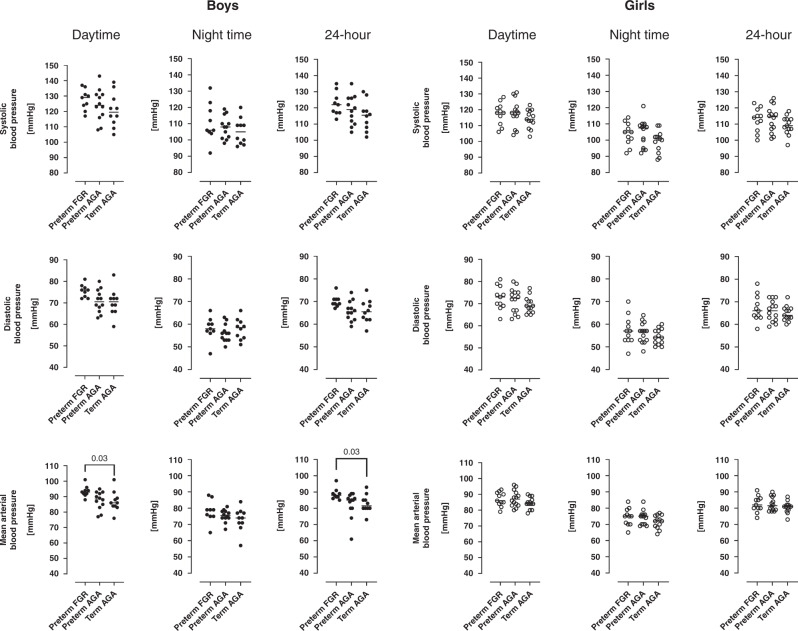
24-hour ambulatory blood pressure measurements in boys (left panel) and girls (right panel). *Upper row* shows systolic blood pressures, *middle row* shows diastolic blood pressures and *lower row* shows mean arterial blood pressure. *Left panel* shows daytime blood pressures, *middle panel* show nighttime blood pressures and *right panel* shows 24-hour blood pressures in adolescents born preterm with fetal growth restriction (preterm FGR), preterm with birth weight appropriate for gestational age (preterm AGA), and adolescents born at term (term AGA). For boys, median daytime and 24-hour mean arterial blood pressures were higher in preterm FGR compared to the term AGA group ((93 (88–101 mmHg) vs. 86 (76–101 mmHg); *p* = 0.03) and (88 (85–97 mmHg) vs. 82 (73–93 mmHg); *p* = 0.03, respectively)). FGR fetal growth restriction, AGA birth weight appropriate for gestational age. Lines indicate median.

Figure 3 and Tables 1, 3 show blood pressure data. There were no group differences in systolic (*p* = 0.37) or diastolic (*p* = 0.08) office blood pressure measurements (Table 1) and no difference observed after stratifying for sex (all *p* ≥ 0.38). There were no differences in the prevalence of prehypertension, hypertension, or pathological day-to-night ratio in mean arterial blood pressure between groups (all *p* ≥ 0.1) (Table 3).

**Table 3:** 24-hour ambulatory blood pressure measurements.

	Preterm FGR	Preterm AGA	Term AGA	*P* between groups
	*n* = *20*	*n* = *27*	*n* = *23*	
Daytime				
Systolic blood pressure				
Prehypertensive (*n* (%))	1 (5%)	2 (7%)	0	0.63
Hypertensive (*n* (%))	2 (10%)	4 (15%)	1 (4%)	0.48
Diastolic blood pressure				
Prehypertensive (*n* (%))	2 (10%)	1 (4%)	1 (4%)	0.68
Hypertensive (*n* (%))	0	0	0	N/A
Nighttime				
Systolic blood pressure				
Prehypertensive (*n* (%))	2 (10%)	0	0	0.08
Hypertensive (*n* (%))	3 (15%)	1 (4%)	0	0.1
Diastolic blood pressure				
Prehypertensive (*n* (%))	1 (5%)	0	0	0.29
Hypertensive (*n* (%))	1 (5%)	0	1 (4%)	0.52
Nocturnal dip (<10%)	3 (15%)	3 (11%)	2 (9%)	0.9

Data are presented as number (%).

*FGR* fetal growth restriction, *AGA* birth weight appropriate for gestational age, *N/A* not applicable.

There was no difference in systolic blood pressure between groups for either sex as determined by 24-hour ABPM (all *p* ≥ 0.21). In boys, the preterm FGR group had higher daytime mean arterial blood pressure (93 mmHg vs. 86 mmHg; *p* = 0.03) and 24-hour mean arterial blood pressure (88 vs. 82 mmHg; *p* = 0.03) as compared to the term AGA group. Further, in boys, trend analyses showed increasing diastolic and mean arterial blood pressures from term AGA to preterm AGA to preterm FGR for 24-hour and for daytime blood pressure (all *p* ≤ 0.03) (Fig. 3). In girls, no group differences or trends were observed in any blood pressure variables (all *p* ≥ 0.1) (Fig. 3).

### Arterial stiffness

Table 4 shows blood flow measurements, aortic centerline distance and cross-sectional area of the ascending aorta and descending aorta at diaphragm level. There were no differences in these variables after correction for BSA between groups (all *p* ≥ 0.06).

**Table 4:** Magnetic resonance imaging variables.

				*P*
	Preterm FGR	Preterm AGA	Term AGA	Between all groups
	*n* = *22*	*n* = *22*	*n* = *26*	
Stroke volume (ml)	68 (53–104)	74 (52–115)	81 (54–121)	0.08
Cardiac output (L/min)	5.2 (2.8–7.4)	5.8 (3.1–9.2)	6.1 (4.0–9.5)	0.3
Cardiac index (L/min/m²)	3.4 (1.9–4.9)	3.6 (2.2 –5.5)	3.6 (2.6–5.2)	0.7
Aortic distance (3D angiography) (mm)	170 (150–227)^**(*p*** **=** **0.02**)^	190 (149–258)	193 (153–237)	**0.02**
Aortic distance (3D angiography) (mm/m²)	112 (87–149)	116 (94–169)	116 (86–150)	0.8
Ascending aorta				
Minimal cross-sectional area (cm²)	3.4 (2.5–4.7)^**(*p*** = **0.02**)^	3.5 (2.6–5.5)	4 (3.0–5.7)	**0.01**
Minimal cross-sectional area (cm²/m²)	2.2 (1.5–2.9)	2.1 (1.5–3.2)	2.4 (1.8–3.3)	0.1
Maximal cross-sectional area (cm²)	5.0 (3.6–6.5)^**(*p*** = **0.02**)^	5.1 (3.8–7.7)	5.8 (4.6–8.4)	**0.01**
Maximal cross-sectional area (cm²/m²)	3.1 (2.4–4.2)	3.1 (2.6–4.5)	3.5 (2.7–4.9)	0.08
Descending aorta				
Minimal cross-sectional area (cm²)	1.1 (0.7–1.8)	1.1 (0.8–1.7)	1.3 (0.8–2.4)	0.1
Minimal cross-sectional area (cm²/m²)	0.7 (0.5–1.2)	0.7 (0.5–1.0)	0.8 (0.6–1.3)	0.2
Maximal cross-sectional area (cm²)	1.7 (1.3–2.6)^**(*p*** =** 0.01**)^	1.9 (1.3–2.6)	2.0 (1.3–3.5)	**0.02**
Maximal cross-sectional area (cm²/m²)	1.1 (0.9–1.7)	1.2 (0.8–1.5)	1.2 (1.0–1.8)	0.06
Arterial stiffness				
Pulse wave velocity (m/s)	3.7 (3.2–5.1)	3.7 (3.1–4.6)	3.6 (3.1–5.0)	0.7
Distensibility ascending aorta (10^−3^ mmHg^−1^)	9.4 (4.4–14.7)	9.4 (6.6–13.9)	9.4 (6.0–12.5)	>0.9
Distensibility descending aorta (10^−3^ mmHg^−1^)	9.4 (4.8–17.9)	11.3 (5.9–16.7)	10.6 (7.0–17.7)	0.5

Data are presented as median (range). Differences were only found between preterm FGR and term AGA. Bold indicates differences between groups.

*FGR* fetal growth restriction, *AGA* birth weight appropriate for gestational age.

**Fig. 4:**
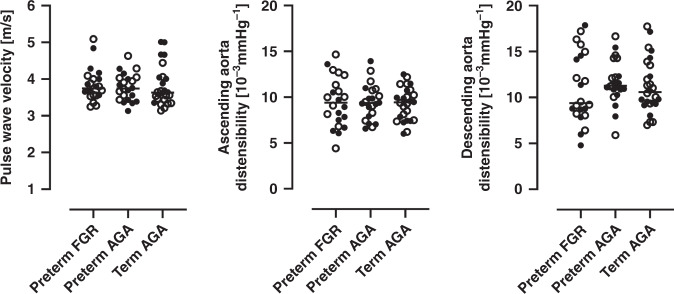
Thoracic aortic pulse wave velocity and aortic distensibility. *Left graph* shows pulse wave velocity, *middle graph* shows distensibility in the ascending aorta and *right graph* shows distensibility in the descending aorta at diaphragm level. Closed circles indicate boys and open circles indicate girls. For girls, median pulse wave velocity was higher in preterm AGA group as compared to the term AGA group (3.9 m/s vs. 3.5 m/s; *p* = 0.04). FGR fetal growth restriction, AGA birth weight appropriate for gestational age. Lines indicate median.

Figure 4 and Table 4 shows PWV and aortic distensibility. There were no differences in PWV or distensibility in the ascending aorta or the descending aorta at diaphragm level between groups (all *p* ≥ 0.5). When stratifying for sex, median PWV was higher in girls in the preterm AGA group as compared to girls in the term AGA group (3.9 m/s vs. 3.5 m/s; *p* = 0.04).

Interobserver variability for aortic centerline distance and PWV, reported as median (95% LoA; i.e., 2.5th to 97.5th percentile), were −6 (−2 to −12) mm and −0.2 (−0.6 to 0.02) m/s, respectively. Median aortic centerline distance between observes was 195 mm whereas median PWV was 3.9 m/s.

### Cystatin C

Median cystatin C-based eGFR were for the preterm FGR group 94 [IQR 78–109] ml/min/1.73 m^2^, for the preterm AGA group 100 [IQR 85–118] ml/min/1.73 m^2^ and for the term AGA group 100 [IQR 89–110] ml/min/1.73 m^2^ (*p* = 0.35).

## Discussion

The current study shows that very preterm birth due to early onset fetal growth restriction is associated with subtle blood pressure increases in adolescent boys. Further, arterial stiffness was increased in girls born very preterm with appropriate birth weight. Renal function did not differ between groups.

### Blood pressure

There were no group differences in prevalence of prehypertension or hypertension as determined by 24-hour ABPM in adolescence. Similar observations, also using 24-hour ABPM, have been noted in a recent cohort of extremely preterm children (~8 years old) born between 2008 and 2011,^11^ however the effect of FGR or birth weight deviation on blood pressure was not studied. As in the current study, kidney function was normal in the previous study,^11^ indicating a limited negative effect of very and extremely preterm birth on kidney function and blood pressure in childhood and adolescence.

In absolute numbers, with no adjustment for age or height, there were no differences in systolic blood pressure for either daytime or nighttime for either sex. However, boys born preterm FGR had higher diastolic and mean arterial blood pressures in comparison with boys in the term AGA group. This is partly in line with the nation-wide follow-up study of the ’Extremely Preterm Infants in Sweden Study’ (EXPRESS) cohort,^12^ which showed that boys but not girls have higher but still normal systolic and diastolic office blood pressure compared to controls. The current study, using 24-hour ABPM, indicates an additive effect of FGR to that of very preterm birth on blood pressure in boys. The ‘EXPRESS’ study lacked the definition of true FGR as the study did not have access to fetal blood flow velocity measurements. Further, the ‘EXPRESS’ study used office blood pressure, which in the current study showed no difference between groups, as opposed to the observed differences based on 24-hour ABPM. Trends in higher blood pressures for boys born preterm FGR as compared to preterm AGA may be due to differences in peri- and neonatal morbidity as both maternal hypertension and the presence of bronchopulmonary dysplasia, with higher prevalence in the preterm FGR group, are associated with increased blood pressure and arterial stiffness.^31–33^

The current and other recent studies.^11,12^ indicate that preterm birth associates with higher but still normal blood pressure in childhood and adolescence, with male sex and FGR as possible exacerbating factors. Earlier studies show strong associations between preterm birth and hypertension,^3,15,34^ with a possible lessened effect of preterm birth on blood pressure in those born after 1990.^34^ These earlier studies, in contrast to the current study and more recent studies, suggest female sex as an exacerbating factor.^15^ Whether the current and other recent cohorts, born in late 1990s and later, continues to show a limited and sex-specific effect of preterm birth and FGR on blood pressure when reaching adulthood remains to be studied. Nevertheless, even a small increase in blood pressure as observed for boys in the preterm FGR group in the current study, may have negative effects on future incidence of cardiovascular disease.^35,36^

Although not significantly different, maternal smoking was almost three times as common in the preterm AGA group as compared to the preterm FGR group and could be hypothesized to be associated with increased blood pressure in this group.^37^ Other factors indicative of socioeconomic differences,^38^ such as parental educational status and cohabitation in childhood that are known to impact blood pressure,^39^ were similar between groups. Socioeconomic factors could thus not explain blood pressure differences between groups in the current study.

Further, the differences found using 24-hour ABPM were not observed using standard office blood pressure, underscoring the increased sensitivity of 24-hour ABPM in the assessment of future cardiovascular risk after very preterm birth and FGR.

### Arterial stiffness

The current study showed that PWV and aortic distensibility were similar between groups, irrespective of preterm birth and FGR. This is in contrast to earlier studies indicating increased arterial stiffness after either preterm birth or low birth weight.^4,5,40,41^ Whether low birth weight due to FGR exacerbates the effect of preterm birth is not clear from these earlier studies. Contrary to Cheung et al.,^41^ the current study showed no differences in arterial stiffness between groups and thus no additive effect of FGR to that of very preterm birth. More in line with the current study, however, the recent ‘EXPRESS’ studies showed normal office blood pressure,^12^ lower carotid stiffness, and no increase in aortic stiffness assessed by ultrasonography.^13^ in children born extremely preterm compared to those born at term.

When stratifying for sex, PWV was higher for girls in the preterm AGA group as compared to girls in the term AGA group. Increased arterial stiffness has previously been shown in adolescent girls born very or extremely preterm with no additional effect of low birth weight for gestational age to that of preterm birth.^5^ In contrast to that previous study, however, the current study population had normal blood pressure, possibly indicating improved cardiovascular outcome in this more recent study population.

Finally, both the ascending and descending aorta were smaller in the preterm FGR group as compared to the term AGA group. Although not significant after BSA adjustment, most likely due to sample size, a visual trend of smaller vessels was observed for both groups born very preterm. A smaller and stiffer arterial tree increases afterload and is hypothesized to predispose for hypertension and cardiovascular disease both in those born preterm and after FGR.^42^ Differences in arterial stiffness and aortic dimensions between the preterm groups in the current study suggest differences in vascular development and subsequent response to extrauterine life. Whether differences in aortic dimensions or arterial stiffness are indicative of future cardiovascular disease in the current population remain to be shown.

### Limitations

Not all individuals from the original cohort participated in the current study, limiting statistical power. However, the strict inclusion criteria and close matching between groups and minimal differences in peri- and neonatal data observed between those participating and those who opted out, limit the potential negative effects of the small sample size. Finally, puberty may affect results and onset of puberty was not determined. It is however likely that most if not all participants were in puberty.^43^

## Conclusions

Very preterm birth due to early onset fetal growth restriction is associated with higher, yet normal blood pressure in adolescent boys. This difference suggests an existing but limited impact of very preterm birth on cardiovascular risk in adolescence, possibly enhanced by male sex and fetal growth restriction.

## Data Availability

Anonymized raw and/or analyzed data are available from the corresponding author on reasonable request.
